# MIsoMine: a genome-scale high-resolution data portal of expression, function and networks at the splice isoform level in the mouse

**DOI:** 10.1093/database/bav045

**Published:** 2015-05-07

**Authors:** Hong-Dong Li, Gilbert S. Omenn, Yuanfang Guan

**Affiliations:** ^1^Department of Computational Medicine and Bioinformatics, ^2^Department of Internal Medicine and ^3^Department of Electrical Engineering and Computer Science, University of Michigan, Ann Arbor, MI 48109, USA

## Abstract

Products of multiexon genes, especially in higher organisms, are a mixture of isoforms with different or even opposing functions, and therefore need to be treated separately. However, most studies and available resources such as Gene Ontology provide only gene-level function annotations, and therefore lose the differential information at the isoform level. Here we report MIsoMine, a high-resolution portal to multiple levels of functional information of alternatively spliced isoforms in the mouse. This data portal provides tissue-specific expression patterns and co-expression networks, along with such previously published functional genomic data as protein domains, predicted isoform-level functions and functional relationships. The core utility of MIsoMine is allowing users to explore a preprocessed, quality-controlled set of RNA-seq data encompassing diverse tissues and cell lineages. Tissue-specific co-expression networks were established, allowing a 2D ranking of isoforms and tissues by co-expression patterns. The results of the multiple isoforms of the same gene are presented in parallel to facilitate direct comparison, with cross-talking to prioritized functions at the isoform level. MIsoMine provides the first isoform-level resolution effort at genome-scale. We envision that this data portal will be a valuable resource for exploring functional genomic data, and will complement the existing functionalities of the mouse genome informatics database and the gene expression database for the laboratory mouse.

**Database URL**: http://guanlab.ccmb.med.umich.edu/misomine/

## Introduction

Alternative splicing is a fundamental process in multicellular organisms, through which a single gene produces multiple transcripts. The resulting protein isoforms may have large differences in their 3D-structures, expression patterns, tissue specificity and biological functions (1–4). Deciphering these differences is essential to precise understanding of gene functions and their relevance to phenotypes and diseases (5–8). Recent accumulation of RNA-seq data and state-of-the-art analytic tools, including but not limited to TopHat (9), Cufflinks (10) and Sailfish (11), have made the estimation of expression levels at the isoform-level possible. Such advancement has stimulated functional genomic data integration at the isoform level (2). Such progress calls for the establishment of a data portal that provides tissue-specific expression and co-expression patterns analogous to what have been established at the gene level (12–16).

We have developed MIsoMine, a data portal that serves functional genomic data analysis for isoforms in the mouse system. This effort is rooted in our interaction with several labs at the Jackson Laboratory; we envision that this portal will benefit the mouse genetics community. Mouse is the primary model organism used in experimental studies of alternatively spliced isoforms. MIsoMine provides curated and quality-controlled meta-data from RNA-seq expression and tissue-specific co-expression networks through an interactive web interface. The isoform expression was estimated using the Sailfish software (11), which is a computationally extremely efficient method and also has high accuracy. MIsoMine is connected to sequence information in RefSeq (17), transcript structure in UCSC genome browser (18), domains in Pfam (19) and provides the predicted functions and functional relationship networks at the splice isoform level based on our recent work (1, 2, 20, 21). Such co-expression network, function and functional relationship predictions are available at the gene level (12–15, 22), including several resources established by us (16, 23, 24), but they are not yet available at the isoform level for the mouse.

The functionality of MIsoMine complements that of several existing databases for the mouse. The gene expression database (GXD) (25) hosted by the Jackson laboratory, e.g. provides a rich and carefully curated resource for multiple types of mRNA and protein expression information, including data from RNA *in situ* hybridization, immunohistochemistry, *in situ* reporter (knock in), reverse transcriptase-polymerase chain reaction, northern blot and western blot experiments (26). GXD’s primary focus is not isoform-level expression analysis through RNA-seq. At the sequence level, ENSEMBL provides sequence and domain information for splice isoforms of a wide spectrum of species including human and mouse. The alternative splicing database focuses on splicing events computationally delineated from Expressed Sequence Tags (EST)/cDNA data and has been integrated into ENSEMBL (27). In the Alternative Splicing Annotation Project (ASAP) database, exon–intron structure, tissue specificity and protein isoform sequences resulting from alternative splicing in human are provided (28). At the functional genomics level, databases are primarily concerned with human, but not mouse. For example, the DBATE is a database of RNA-seq based expression of alternative transcripts in 13 normal and diseased tissues in the human (29). Similarly, the RNA-seq atlas database provides RNA-seq expression profiles of human transcripts (30). The APPRIS focuses on selecting one principal isoform for each multiexon gene based on structural and evolutionary information between species (31). Complementing the above resources, MIsoMine provides a portal for exploring mouse functional genomic data, and is intended to serve the mouse genetics community.

## Data content

**Figure 1.**
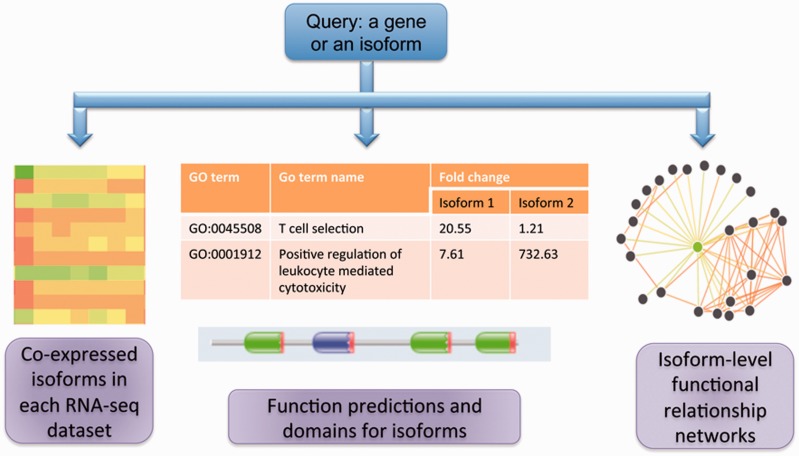
The schematic of the MIsoMine data portal. It contains multiple levels of functional genomic information including expression and co-expression networks. It is linked to external information about domains and sequences and provides predicted functions and functional relationships at the splice isoform level in the mouse.

Figure 1 summarizes the core functionalities in MIsoMine: (i) Meta-data for isoform-level expression values and co-expression networks for specific tissues and cell lineages. (ii) Isoform-level function predictions and functional relationship predictions. (iii) Links to sequence and protein domain information. A statistical summary of the MIsoMine data portal is provided in Table 1.

**Table 1.** The statistics summary of the MIsoMine data portal

Genome and annotation version	Genome assemblyGene annotation	GRCm38RefSeq 38
Gene statistics	Number of Genes	21 697
	Number of protein-coding genes	17 542
	Number of noncoding genes	4155
Isoform statistics	Number of isoforms	49 007
	Number of protein-coding transcripts	42 289
	Number of noncoding transcripts	6718

### Sample quality control

**Figure 2.**
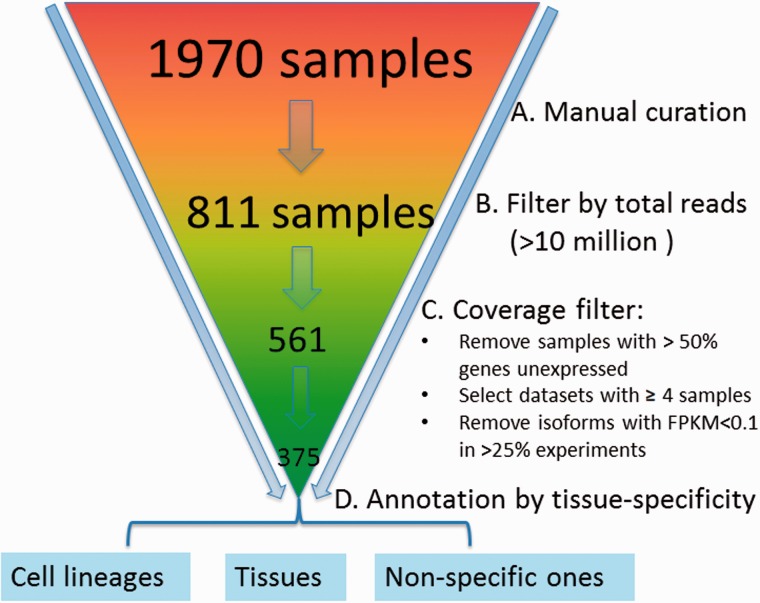
Preprocessing and quality control of RNA-seq data. The initial set of 1970 samples downloaded from the SRA database was first manually examined to ensure that samples have are only from RNA-sequencing of mouse samples followed by removal of those with <10 million reads, resulting in 561 samples. Data are further processed by (i) removing samples with >50% genes unexpressed, (ii) removing datasets with <4 samples, (iii) removing isoforms with RPKM < 0.1 in >25% experiments and (iv) log_2_ transformation; 375 samples were retained finally and grouped into 43 datasets, which are mainly cell lineage- or tissue-specific.

We downloaded an initial set of 1970 samples from the sequence read archive (SRA) database (32) for the mouse (Figure 2). A sample refers to a cell line or a tissue specimen for which RNA-seq data have been measured. A collection of samples generated from the same study form a dataset. We manually curated them by removing samples that were not really from RNA-seq (but contained the keyword ‘RNA-seq’ in the initial downloaded files) or contained data only from other species. After doing so, 811 samples were left. To ensure sufficient coverage of the majority of the isoforms, the samples with <10 million reads were further removed, resulting in 561 samples. Because correlation coefficients based on three or fewer samples are noisy, datasets with <4 samples were removed. Finally, we obtained 375 samples falling into 43 datasets.

### RNA-seq expression quantification

For the remaining 375 samples, we downloaded their raw sequence data from SRA and converted them to fastq format. Then, the transcript expression values were calculated using the Sailfish software (11), which takes as input the fastq file and the transcript sequence from the most recent RefSeq transcriptome annotation based on the GRCm38 genome build (ftp://ftp.ncbi.nlm.nih.gov/genomes/M_musculus/GFF/ref_GRCm38.p2_top_level.gff3.gz). Briefly, Sailfish is an extremely fast alignment-free method for quantifying transcripts (11), with similar accuracy as for example the Cufflinks pipeline (10). The expression is quantified in terms of reads per kilobase of exon per million (RPKM fragments mapped). Within each dataset, isoforms with RPKM < 0.1 in more than one-fourth of the samples were removed. Then, expression values were log_2_-transformed for subsequent analysis according to the practice in Cuffdiff (10).

### Constructing co-expression networks at the isoform level for the mouse

Assuming that the *i*th (*i* = 1, 2, … 43) dataset contained k*_i_* isoforms, we calculated the Pearson correlation coefficients between all possible splice isoform pairs, resulting in a correlation matrix **C*_i_*** of size *k_i_* × *k_i_*. **C***_i_* can be graphically displayed as a co-expression network by treating isoforms as nodes and correlation coefficients between two isoforms as edges. In total, there were 43 co-expression networks. Further, based on our previous work (16, 33, 34), the correlation coefficients in each dataset were Fisher’s *z*-transformed in order to be normally distributed and comparable across datasets using the following formula (35):

(1) $$z=\frac{1}{2}\ln\left(\frac{1+r}{1-r}\right)$$

where *r* stands for the original correlation coefficient and *z* represents the transformed value. Then, for a specific isoform pair, the average correlation over all datasets where it occurs was calculated. This mean correlation network was used for ranking isoforms. According to the tissue information, 38 datasets are tissue- or cell lineage-specific, such as focusing on the heart or capturing different time points during neuronal differentiation of embryonic stem cells. Another four are a mixture of tissues or cell lines, which represent variations across different body compartments. For example, the dataset SRP007412 included different tissues such as brain, liver, testis, heart, kidney and cerebellum. One dataset that had no information but passed the quality control pipeline was also included.

### Query system and genes/alias mapping

**Figure 3.**
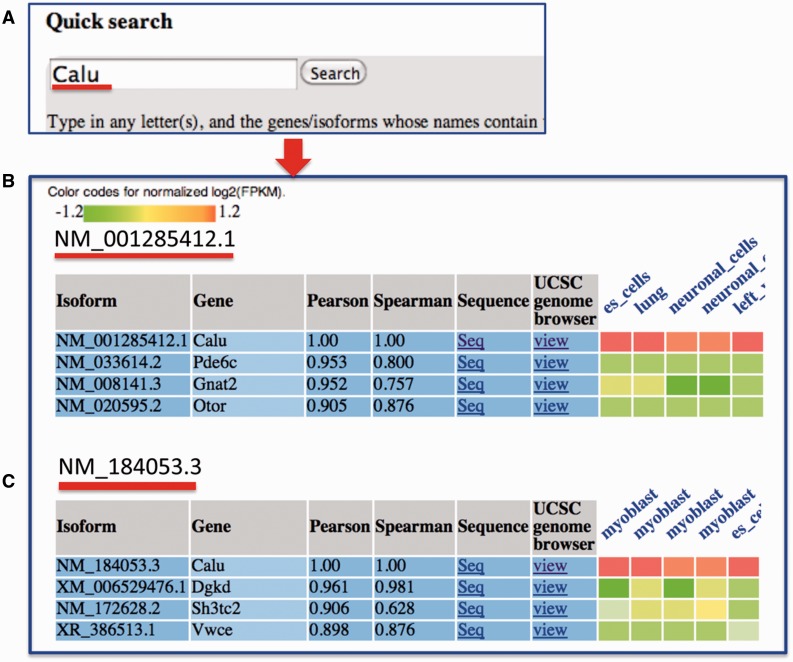
An example search for MIsoMine data portal from the ‘expression’ page and the returned results. (**A**) Querying the *Calu* (calumenin) gene. For illustration, (**B**) and (**C**) represent the normalized expression heat map of the top three correlated isoforms of NM_001285412.1 and NM_184053.3 of the *Calu* gene, respectively. In the heatmap in B and C, each column represents one sample and the tissue information is labeled on the top of each column. Original expression data of the top correlated isoforms can be retrieved from the webpage.

In the query system, for each isoform, its correlations with all the other isoforms in the mean co-expression network are sorted in descending order, and a co-expression sub-network containing only the top isoforms is identified. Then, the RNA-seq expression values of the top co-expressed isoforms, which were normalized for each dataset, are shown as a heat map (Figure 3). We calculated the Spearman rank correlation and presented it in the heat map. Additionally, the top datasets, in which these isoforms are highly co-expressed, are presented. This function helps users analyse the tissues or cell lineages in which the isoforms are likely to be co-expressed. The entire query-specific analysis of expression results is downloadable as tab-delimited files from the website.

Due to the fact that a gene may contain one or more alias, using only the gene symbols that were included in the mouse gene annotation file would not be sufficient. Therefore, we extracted the mouse genes and their alias information from the NCBI ftp site (ftp://ftp.ncbi.nlm.nih.gov/gene/DATA/GENE_INFO/Mammalia/Mus_musculus.gene_info.gz) and found that 20 597 genes have 86 939 alias names in total. These alias data are stored in our database, allowing users to query by alias.

### Links to relevant information in existing databases and functional genomics information for mouse isoforms

**Figure 4.**
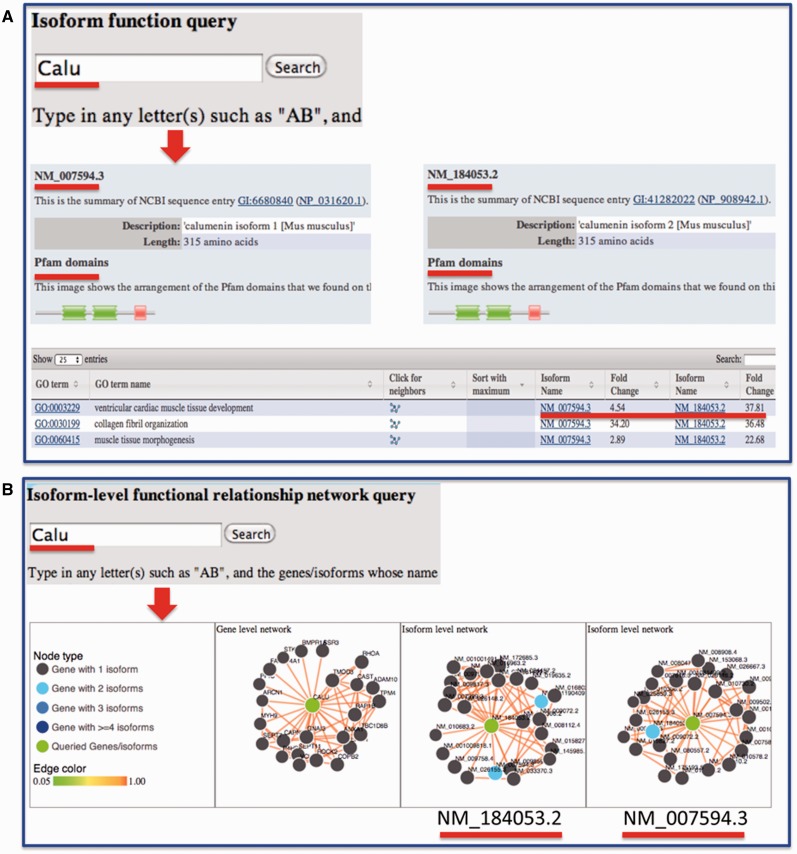
Example query from the MIsoMine ‘Function’ and ‘Network’ pages. (**A**) Query the isoform-level function database using *Calu* (calumenin) as an example. While having the same number of amino acids, its two isoforms NM_007594.3 and NM_184053.2 are different in their exons. NM_184053.2 lacks the exon in NM_007594.3 (chr6:29,361,294-29,361,487) but contains an additional exon (chr6:29,361,560-29,361,753). The Pfam domains of the two isoforms are shown. The fold change of probabilities with respect to random predictions for the two isoforms to be annotated to a function (GO term) is displayed in the table. (**B**) Query the isoform-level functional relationship network database using *Calu* as an example. In this network, a node is an isoform and an edge indicates the probability that two isoforms work in the same biological process or pathway. This network contains the functional relationships of all possible mouse isoform pairs. The top linked isoforms for each query are displayed using the *d3* software.

Sequence information for each isoform was retrieved from RefSeq and made available through the website. We also linked our data portal to the UCSC genome browser so that the transcript structure can be visualized. We previously developed a suite of algorithms for predicting isoform-level functions and functional relationships through integrating RNA-seq data and other publicly available functional genomic data (1, 2, 20, 21). For each isoform, the predicted functions are provided and can be compared with other isoforms from the same gene (Figure 4). Domain information for splice isoforms, which are publicly available from Pfam database (19), was integrated for investigating structural differences among isoforms. In sum, functions and functional relationships at the isoform level are provided for each isoform in the mouse.

## Usage

The MIsoMine portal is open-source through a convenient, interactive website, with search options and help documents available for making queries and understanding the output. On the ‘Home’ page, we implemented a common query box that allows users to search expression, function and network for isoforms. For example, one can input gene symbols like *Calu* (calumenin) or isoform names like NM_007499.2 and click ‘Search’ to be guided to RNA-seq expression, isoform functions and functional relationship networks. ‘Autocomplete’ functions were implemented for all query boxes throughout the website. These example gene/isoform queries and the ‘Autocomplete’ function conveniently allow users who are not familiar with gene/isoform names to make a trial search. For each isoform of the query gene, its top correlated isoforms are listed in descending order (Figure 3C). In this list, one can click the ‘Seq’ or ‘view’ links to investigate the sequence or transcript structure information of the top-connected isoform for a specific isoform. The RNA-seq expression values in terms of unit length-normalized log_2_-transformed RPKM are shown as a heat map. Top datasets, in which the returned isoforms are most correlated, are displayed. Users may download the expression across all the 375 samples through the link right above the heat map.

For users who wish to investigate the potential functions and functional relationships of mouse isoforms, they may directly input a gene or an isoform on the ‘Function’ or ‘Network’ page. Taking the *Calu* gene as an example (Figure 4A), the protein length and domain information extracted from the Pfam database for each of its isoforms will be displayed. In the table later, the fold change, which is the ratio of the predicted score for an isoform to be annotated to a Gene Ontology (GO) biological process to that by chance, is presented. Comparing fold change values reveals how functionally different the isoforms from the same gene could be. To ensure that biological functions are not too specific and not too general, only GO terms annotated with >20 and <300 genes are considered. From the ‘Network’ page, users may obtain and visualize the functional relationship network at the gene and splice isoform level for their queried genes or isoforms of interest. In the isoform-level functional network, each node is an isoform and each edge represents the probability that two isoforms co-function in the same biological process or pathway (21). For instance, after querying the *Calu* gene, three networks will be shown (Figure 4B), with one at the gene level and two at the isoform level. For multi-isoform genes, we mapped the top connected interactors of each isoform to genes followed by calculating the number of shared genes between isoform and gene-level networks. We found that, of the top 25 connections as shown in the website, gene-level and isoform-level networks have on average 12 shared connections, which is much smaller than the baseline of 25 obtained by assuming that there is no difference between gene and isoform-level networks. This implies a big difference between isoform- and gene-level networks.

In addition, MIsoMine provides technical support to its users through a user support staff and documentation. User support can be contacted via email.

## Conclusions

The products of multiexon genes are a mixture of splice isoforms carrying different or even opposing functions. It is therefore essential to investigate gene properties specific to individual isoforms. We have developed an integrated portal that encompasses domain, expression, functions and networks at the splice isoform level, allowing for more precise understanding of genes compared with traditional approaches that treat gene as a single entity. Our proposed method is generic and can be readily extended to other species such as human, as we are doing. We expect that MIsoMine will become a unique resource towards an isoform-centric view of mouse genome for the genetics community.

## Funding

This work is supported by National Institutes of Health grants 1R21NS082212-01 (YG) and U54ES017885 (GSO). Funding for open access charge: National Institutes of Health 1R21NS082212-01 (YG).

*Conflict of interest*. None declared.
